# Supplementary material for: Family-Level Multimorbidity among Older Adults in India: Looking through a Syndemic Lens
Source: Int J Environ Res Public Health. 2022 Aug 10;19(16):9850. doi: 10.3390/ijerph19169850 (PMC9408391; doi:10.3390/ijerph19169850)
Supplement: Supplementary file 1 [file ijerph-19-09850-s001.zip › ijerph-1816581-supplementary.pdf]

Table S1. Family level Multimorbidity (N = 22,526 families).

No. of family membershaving multimorbidity	No of families(n)	Percentage (%)
None	5254	23.32
One	7256	32.21
Two	9123	40.50
Three	748	3.32
Four or more	145	0.64
